# Age, Sex, and Cerebral Microbleeds Affect White Matter Integrity Across Adulthood After Mild Traumatic Brain Injury

**DOI:** 10.1093/geroni/igab046.3021

**Published:** 2021-12-17

**Authors:** David Robles, Ammar Dharani, Nikhil Chaudhari, Kenneth Rostowsky, Layal Wehbe, Michelle Ha, Van Ngo, Andrei Irimia

**Affiliations:** 1 University of Southern California, Los Angeles, California, United States; 2 University of Southern California, University of Southern California, California, United States

## Abstract

The contributions of age, sex, and cerebral microbleeds (CMBs) to WM changes after mild traumatic brain injury (mTBI) have not been studied. We used diffusion tensor imaging (DTI) to map WM fractional anisotropy (FA) changes across the first ~6 months post-mTBI in 109 subjects aged 18-77 (46 females; age µ: 40 y, σ: 17 y) imaged within ~1 week post-injury and ~6 months later. After partialing out age, sex, and CMB counts, significant mean FA decreases were found in the anterior body, posterior body, and splenium of the corpus callosum (CC; p = 0.003, 0.009 and 0.015, respectively), left superficial frontal fasciculus (p = 0.008), and left branch of the corticospinal tract (CST; p = 0.007). Age contributed to mean FAs measured acutely in the CC body (p = 0.04), and chronically in the CC genu (p < 0.001), CC body (p = 0.01), and middle longitudinal fasciculi (p = 0.04), older adults exhibiting larger decreases. CMB counts were positively associated with mean FA decreases in the CC body (p = 0.04) and middle longitudinal fasciculi (p = 0.04). Significant age-by-sex and CMB count-by-age interactions mediated FA decreases in the CC genu (p = 0.02 and p = 0.03, respectively), older males exhibiting larger decreases. Thus, the CC, longitudinal fasciculi, superficial frontal WM and CST are particularly vulnerable to post-traumatic neurodegeneration moderated by age, sex and CMB count, men and older adults being at highest risk for adverse effects. Future research should investigate our findings relative to cognitive function.

